# Update: Interim Guidance for Health Care Providers Evaluating and Caring for Patients with Suspected E-cigarette, or Vaping, Product Use Associated Lung Injury — United States, October 2019

**DOI:** 10.15585/mmwr.mm6841e3

**Published:** 2019-10-18

**Authors:** David A. Siegel, Tara C. Jatlaoui, Emily H. Koumans, Emily A. Kiernan, Mark Layer, Jordan E. Cates, Anne Kimball, David N. Weissman, Emily E. Petersen, Sarah Reagan-Steiner, Shana Godfred-Cato, Danielle Moulia, Erin Moritz, Jonathan D. Lehnert, Jane Mitchko, Joel London, Sherif R. Zaki, Brian A. King, Christopher M. Jones, Anita Patel, Dana Meaney Delman, Ram Koppaka, Lung Injury Response Clinical Working Group, Lung Injury Response Epidemiology/Surveillance Group, Lung Injury Response Clinical Working Group, Anne Griffiths, Annette Esper, Carolyn S. Calfee, Don Hayes, Devika R. Rao, Dixie Harris, Lincoln S. Smith, Scott Aberegg, Sean J. Callahan, Lung Injury Response Epidemiology/Surveillance Group, Rashid Njai, Jennifer Adjemian, Macarena Garcia, Kathleen Hartnett, Kristen Marshall, Aaron Kite Powell, Adebola Adebayo, Minal Amin, Michelle Banks, Jordan Cates, Maeh Al-Shawaf, Lauren Boyle-Estheimer, Peter Briss, Gyan Chandra, Karen Chang, Jennifer Chevinsky, Katelyn Chiang, Pyone Cho, Carla Lucia DeSisto, Lindsey Duca, Sumera Jiva, Charlotte Kaboré, John Kenemer, Akaki Lekiachvili, Maureen Miller, Yousra Mohamoud, Cria Perrine, Mays Shamout, Lauren Zapata, Francis Annor, Vaughn Barry, Amy Board, Mary E. Evans, Allison Gately, Brooke Hoots, Cassandra Pickens, Tia Rogers, Alana Vivolo-Kantor, Alissa Cyrus, Tegan Boehmer, Emily Glidden, Arianna Hanchey, Angela Werner, Shideh Ebrahim Zadeh, Donna Pickett, Victoria Fields, Michelle Hughes, Varsha Neelam, Kevin Chatham-Stephens, Kevin O’Laughlin, Mary Pomeroy, Sukhshant K. Atti, Jennifer Freed, Jona Johnson, Eva McLanahan, Kate Varela, Jennifer Layden, Jonathan Meiman, Nicole M. Roth, Diane Browning, Augustina Delaney, Samantha Olson, Dessica F. Hodges, Raschelle Smalley

**Affiliations:** ^1^National Center for Chronic Disease Prevention and Health Promotion, CDC; ^2^Agency for Toxic Substances and Disease Registry, CDC; ^3^Emory University School of Medicine, Atlanta, Georgia; ^4^National Center for Environmental Health, CDC; ^5^National Center for Immunization and Respiratory Diseases, CDC; ^6^Epidemic Intelligence Service, CDC; ^7^National Center for HIV/AIDS, Viral Hepatitis, STD, and TB Prevention, CDC; ^8^National Institute for Occupational Safety and Health, CDC; ^9^National Center for Emerging and Zoonotic Infectious Diseases, CDC; ^10^National Center on Birth Defects and Developmental Disabilities, CDC; ^11^General Dynamics Information Technology; ^12^National Center for Injury Prevention and Control, CDC.; , Pediatric Pulmonary Medicine, Children’s Minnesota; Emory University; Pulmonary and Critical Care Medicine, University of California, San Francisco; Nationwide Children’s Hospital and The Ohio State University; Department of Pediatrics, Division of Respiratory Medicine; UT Southwestern Medical Center; Intermountain Healthcare; University of Washington and Seattle Children’s Hospital; University of Utah; Office of the Director, Deputy Director for Non-Infectious Diseases, CDC; Center for Surveillance, Epidemiology, and Laboratory Services, CDC; National Center for Immunization and Respiratory Diseases, CDC; National Center for Chronic Disease Prevention and Health Promotion, CDC; National Center for Injury Prevention and Control, CDC; Office of Minority Health and Health Equity, CDC; National Center for Environmental Health, CDC; National Center for Health Statistics, CDC; National Center on Birth Defects and Developmental Disabilities, CDC; National Center for Emerging and Zoonotic Infectious Diseases, CDC; Agency for Toxic Substances and Disease Registry, CDC and Emory University School of Medicine; Agency for Toxic Substances and Disease Registry; National Institute for Occupational Safety and Health; Illinois Department of Public Health; Wisconsin Department of Health Services; Eagle Medical Services; Northrop Grumman; G2S Corporation; Student Worksite Program volunteer; Student Worksite Experience Program volunteer

*On October 11, 2019, this report was posted online as an *MMWR *Early Release.*

CDC, the Food and Drug Administration (FDA), state and local health departments, and public health and clinical partners are investigating a multistate outbreak of lung injury associated with the use of electronic cigarette (e-cigarette), or vaping, products. In late August, CDC released recommendations for health care providers regarding e-cigarette, or vaping, product use associated lung injury (EVALI) based on limited data from the first reported cases (*1*,*2*). This report summarizes national surveillance data describing clinical features of more recently reported cases and interim recommendations based on these data for U.S. health care providers caring for patients with suspected or known EVALI. It provides interim guidance for 1) initial clinical evaluation; 2) suggested criteria for hospital admission and treatment; 3) patient follow-up; 4) special considerations for groups at high risk; and 5) clinical and public health recommendations. Health care providers evaluating patients suspected to have EVALI should ask about the use of e-cigarette, or vaping, products in a nonjudgmental and thorough manner. Patients suspected to have EVALI should have a chest radiograph (CXR), and hospital admission is recommended for patients who have decreased blood oxygen (O_2_) saturation (<95%) on room air or who are in respiratory distress. Health care providers should consider empiric use of a combination of antibiotics, antivirals, or steroids based upon clinical context. Evidence-based tobacco product cessation strategies, including behavioral counseling, are recommended to help patients discontinue use of e-cigarette, or vaping, products. To reduce the risk of recurrence, patients who have been treated for EVALI should not use e-cigarette, or vaping, products. CDC recommends that persons should not use e-cigarette, or vaping, products that contain tetrahydrocannabinol (THC). At present, CDC recommends persons consider refraining from using e-cigarette, or vaping, products that contain nicotine. Irrespective of the ongoing investigation, e-cigarette, or vaping, products should never be used by youths, young adults, or women who are pregnant. Persons who do not currently use tobacco products should not start using e-cigarette, or vaping, products.

As of October 8, 2019, 49 states, the District of Columbia, and one territorial health department have reported 1,299 cases of EVALI to CDC, with 26 deaths reported from 21 states (median age of death = 49 years, range = 17–75 years). Among 1,043 patients with available data on age and sex, 70% were male, and the median age was 24 years (range = 13–75 years); 80% were aged <35 years, and 15% were aged <18 years. Among 573 patients who reported information on substances used in e-cigarette, or vaping, products in the 90 days preceding symptom onset, 76% reported using THC-containing products, and 58% reported using nicotine-containing products; 32% reported exclusive use of THC-containing products, and 13% reported exclusive use of nicotine-containing products.* No single compound or ingredient has emerged as the cause of these injuries to date, and there might be more than one cause. Available data suggest THC-containing products play a role in this outbreak, but the specific chemical or chemicals responsible for EVALI have not yet been identified, and nicotine-containing products have not been excluded as a possible cause.

Ongoing federal and state investigations have provided information about the clinical characteristics of cases and a surveillance case definition for confirmed and probable cases has been developed (*1*); this case definition^†^ is not intended to guide clinical care. To inform CDC’s updated interim clinical guidance, on October 2, 2019, CDC obtained individual expert perspectives on the evaluation and treatment of patients with suspected EVALI. Discussions occurred with nine national experts in adult and pediatric pulmonary medicine and critical care who were designated by professional medical societies to participate (Lung Injury Response Clinical Working Group). Evidence supporting CDC’s recommendations include data from medical abstractions reported to CDC, previously published case series (*3*–*5*), and the aforementioned individual expert opinions.

## Clinical Evaluation for Patients with Suspected EVALI

EVALI is considered a diagnosis of exclusion because, at present, no specific test or marker exists for its diagnosis (Box 1). Health care providers should consider multiple etiologies, including the possibility of EVALI and concomitant infection. In addition, health care providers should evaluate alternative diagnoses as suggested by clinical findings and medical history (e.g., cardiac, gastrointestinal, rheumatologic, and neoplastic processes; environmental or occupational exposures; or causes of acute respiratory distress syndrome) (*6*).

BOX 1 **Clinical evaluation for patients with recent history of use of e-cigarette, or vaping, products and suspected lung injury**

**History**

- Ask about respiratory, gastrointestinal, and constitutional symptoms (e.g., cough, chest pain, shortness of breath, abdominal pain, nausea, vomiting, diarrhea, and fever) for patients who report a history of use of e-cigarette, or vaping, products.
- Ask all patients about recent use of e-cigarette, or vaping, products.
  - Types of substances used (e.g., tetrahydrocannabinol [THC], cannabis [oil, dabs], nicotine, modified products or the addition of substances not intended by the manufacturer); product source, specific product brand and name; duration and frequency of use, time of last use; product delivery system, and method of use (aerosolization, dabbing, or dripping).

**Physical exam**

- Assess vital signs and oxygen saturation via pulse-oximetry.

**Laboratory testing**

- Infectious disease evaluation might include
  - Respiratory viral panel including influenza testing during flu season, *Streptococcus pneumoniae*, *Legionella pneumophila*, *Mycoplasma pneumoniae*, endemic mycoses, and opportunistic infections.
- Initial laboratory evaluation
  - Consider complete blood count with differential, liver transaminases, and inflammatory markers (e.g., erythrocyte sedimentation rate and C-reactive protein).
  - In all patients, consider conducting urine toxicology testing, with informed consent, including testing for THC.

**Imaging**

- Chest radiograph.
- Consider chest computed tomography for evaluation of severe or worsening disease, complications, other illnesses, or when chest x-ray result does not correlate with clinical findings.

**Other considerations**

- Further evaluation of patients meeting inpatient admission criteria might include
  - Consultation with pulmonary, critical care, medical toxicology, infectious disease, psychology, psychiatry, and addiction medicine specialists.
  - Additional testing with bronchoalveolar lavage or lung biopsy as clinically indicated, in consultation with pulmonary specialists.

**Patient history.** Based upon medical chart abstraction data submitted to CDC, 95% (323/339) of patients diagnosed with EVALI initially experienced respiratory symptoms (e.g., cough, chest pain, and shortness of breath), and 77% (262/339) had gastrointestinal symptoms (e.g., abdominal pain, nausea, vomiting, and diarrhea). Gastrointestinal symptoms preceded respiratory symptoms in some patients (*1*–*3*). Respiratory or gastrointestinal symptoms were accompanied by constitutional symptoms such as fever, chills, and weight loss among 85% (289/339) of patients (Table).

**TABLE:** Characteristics of patients (N = 342) with e-cigarette use, or vaping, product use associated lung injury (EVALI),* from national EVALI surveillance reports to CDC — United States, 2019^†^

Characteristic	EVALI patients	
	No. (%)	Total no. used in calculation^§^
**Age, median (range) (yrs)**	22 (13–71)	338
**Symptoms reported**		
Any respiratory	323 (95)	339
Any gastrointestinal	262 (77)	339
Any constitutional^¶^	289 (85)	339
**Vital signs**		
Oxygen saturation <95% while breathing room air	143 (57)	253
Tachycardia (heart rate >100 beats/min)	169 (55)	310
Tachypnea (respiratory rate >20 breaths/min)	77 (45)	172
**Clinical course**		
Admission to intensive care unit	159 (47)	342
Age group (yrs)		
13–17	45 (56)	80
18–24	49 (38)	130
25–50	54 (47)	115
≥51	9 (69)	13
Past cardiac disease**	8 (50)	16
No past cardiac disease	151 (46)	326
**Intubation and mechanical ventilation**	74 (22)	338
Age group (yrs)		
13–17	23 (29)	80
18–24	21 (16)	130
25–50	23 (20)	115
≥51	7 (54)	13
Past cardiac disease**	5 (31)	16
No past cardiac disease	70 (21)	326
**Corticosteroids**	252 (88)	287
**Improved after corticosteroids**	114 (82)	140
**Duration of hospitalization (days)**	**Mean (median)**	**Range**
**Age group (yrs)**		
13–17	6.9 (6)	0–23
18–24	6.2 (5)	0–38
25–50	6.6 (6)	0–40
≥51	14.8 (12)	3–31
Past cardiac disease	8.9 (4)	3–31
No past cardiac disease	6.6 (5)	0–40
**Average hospital stay**	6.7 (5)	0–40

**Abbreviation:** E-cigarette = electronic cigarette.

* For cases that had full medical chart abstraction data available.

^†^ Surveillance data through October 3, 2019, from the following 29 U.S states: Alabama, Delaware, Georgia, Hawaii, Idaho, Illinois, Indiana, Iowa, Kansas, Kentucky, Maine, Maryland, Minnesota, Mississippi, Missouri, Montana, Nevada, New Jersey, New Mexico, Oklahoma, Oregon, Rhode Island, South Carolina, South Dakota, Texas, Vermont, Washington, West Virginia, and Wisconsin.

^§^ Patients with missing data were excluded from denominators for selected characteristics.

^¶^ Self-reported fever, chills, and unexpected weight loss.

** Heart failure, heart attack, or other heart conditions.

All health care providers evaluating patients for EVALI should ask about the use of e-cigarette, or vaping, products and ideally should ask about types of substances used (e.g., THC, cannabis [oil, dabs], nicotine, modified products or the addition of substances not intended by the manufacturer); product source, specific product brand and name; duration and frequency of use, time of last use; product delivery system, and method of use (aerosolization, dabbing, or dripping). Empathetic, nonjudgmental, and private questioning of patients regarding sensitive information to assure confidentiality should be employed. Standardized approaches should be used for interviewing adolescents. Resources exist to guide patient interviews, including those of adolescents.^§^ In some situations, asking questions over the course of the hospitalization or during follow-up visits might elicit additional information about exposures, especially as trust is established between the patient and clinicians.

**Physical examination.** For patients who report the use of e-cigarette, or vaping, products, physical examination should include vital signs and pulse-oximetry. Tachycardia was reported in 55% (169/310) of patients and tachypnea in 45% (77/172); O_2_ saturation <95% at rest on room air was present for 57% (143/253) of patients reported to CDC (Table), underscoring the need for routine pulse-oximetry. Among patients identified to date, pulmonary findings on auscultation exam have often been unremarkable, even among patients with severe lung injury (personal communication, Lung Injury Response Clinical Working Group, October 2, 2019).

**Laboratory testing.** Laboratory testing should be guided by clinical findings. A respiratory virus panel, including influenza testing during influenza season, should be strongly considered. Additional testing should be based on published guidelines for evaluation of community-acquired pneumonia.^¶^ Infectious diseases to consider include *Streptococcus pneumoniae*, *Legionella pneumophila*, *Mycoplasma pneumoniae*, endemic mycoses, and opportunistic infections; the likelihood of infection by any of these varies by geographic prevalence and patient medical history. Other abnormal laboratory tests reported in patients with EVALI include elevated white blood cell (WBC) count, serum inflammatory markers (C-reactive protein, erythrocyte sedimentation rate [ESR]), and liver transaminases. In a report of initial patients from Illinois and Wisconsin, 87% had a WBC >11,000/mm^3^ and 93% had an ESR >30mm/hr; 50% of patients had elevated liver transaminases (aspartate aminotransferase or alanine aminotransferase >35 U/L) (*3*). However, at this time, these tests cannot be used to distinguish EVALI from infectious etiologies. In all patients, providers should consider conducting, with informed consent, urine toxicology testing, including testing for THC.

**Imaging**. Radiographic findings consistent with EVALI include pulmonary infiltrates on CXR and opacities on chest computed tomography (CT) scan (*1*,*7*). A CXR should be obtained on all patients with a history of e-cigarette, or vaping, product use who have respiratory or gastrointestinal symptoms, particularly when accompanied by decreased O_2_ saturation (<95%). Chest CT might be useful when the CXR result does not correlate with clinical findings or to evaluate severe or worsening disease, complications such as pneumothorax or pneumomediastinum, or other illnesses in the differential diagnosis, such as pneumonia or pulmonary embolism. In some cases, chest CT has demonstrated findings such as bilateral ground glass opacities despite a normal or nondiagnostic CXR (*3*). Among patients with abnormal CXR findings and a clinical picture consistent with EVALI, a chest CT scan might not be necessary for diagnosis. The decision to obtain a chest CT should be made on a case-by-case basis depending on the clinical circumstances.

**Consultation with specialists.** Consultation with several specialists might be necessary to optimize patient management. For patients being evaluated for possible EVALI, consideration should be given to consultation with a pulmonologist, who can help guide further evaluation, recommend empiric treatment, and review the indications for bronchoscopy. The decision to perform bronchoscopy and bronchoalveolar lavage (BAL) to rule out alternative diagnoses such as pulmonary infection should be made on a case-by-case basis. The value of staining BAL cells or fresh lung biopsy tissue for lipid-laden macrophages (e.g., using oil red O or Sudan Black) in the evaluation of EVALI remains unknown. In addition, there should be a low threshold for consulting with critical care physicians, because, based upon data submitted to CDC, 47% (159/342) of patients were admitted to an intensive care unit and 22% (74/338) required endotracheal intubation and mechanical ventilation (Table); critical care physicians should be consulted to determine optimal management of respiratory failure. Consultation with medical toxicology, infectious disease, psychology, psychiatry, addiction medicine, and other specialists should be considered as warranted by patient circumstances.

## Management of Patients with Suspected EVALI

**Admission criteria and outpatient management.** Several factors should be considered when deciding whether to admit a patient with potential EVALI to the hospital (Box 2). Among 1,002 cases reported to CDC with available data as of October 8, 96% of patients were hospitalized. Patients with suspected EVALI should be admitted if they have decreased O_2_ saturation (<95%) on room air, are in respiratory distress, or have comorbidities that compromise pulmonary reserve. Consider modifying factors such as altitude to guide interpretation of measured O_2_ saturation.

BOX 2 **Management of patients with suspected e-cigarette, or vaping, product use associated lung injury (EVALI)**

**Admission criteria and outpatient management**

- Strongly consider admitting patients with potential lung injury, especially if respiratory distress present, have comorbidities that compromise pulmonary reserve, or decreased (<95%) O_2_ saturation (consider modifying factors such as altitude to guide interpretation).
- Outpatient management for patients with suspected lung injury who have less severe injury might be considered on a case-by-case basis.

**Medical treatment**

- Consider initiation of corticosteroids.
- Early initiation of antimicrobial coverage for community-acquired pneumonia should be strongly considered in accordance with established guidelines.*
- Consider influenza antivirals in accordance with established guidelines.^†^

**Patients not admitted to hospital**

- Recommend follow-up within 24–48 hours to assess and manage possible worsening lung injury.
- Outpatients should have normal oxygen saturation, reliable access to care and social support systems, and be instructed to promptly seek medical care if respiratory symptoms worsen.
- Consider empiric use of antimicrobials and antivirals.

**Post-hospital discharge follow-up**

- Schedule follow-up visit no later than 1–2 weeks after discharge that includes pulse-oximetry testing.
- Consider additional follow-up testing including spirometry and diffusion capacity testing, and consider repeat chest radiograph in 1–2 months.
- Consider endocrinology consultation for patients treated with high-dose corticosteroids.

**Cessation services and preventive care**

- Strongly advise patients to discontinue use of e-cigarette, or vaping, products.
- Provide education and cessation assistance for patients to aid nicotine addiction and treatment or referral for patients with marijuana-use-disorder.^§^
- Emphasize importance of routine influenza vaccination.^¶^
- Consider pneumococcal vaccine.**

* https://www.atsjournals.org/doi/full/10.1164/rccm.201908-1581ST#readcube-epdf; https://academic.oup.com/cid/article/53/7/e25/424286/.

^†^ https://www.cdc.gov/flu/professionals/antivirals/summary-clinicians.htm; https://www.idsociety.org/practice-guideline/influenza/.

^§^ Substance Abuse and Mental Health Services Administrations treatment locator (https://www.samhsa.gov/find-treatment)to find treatment in your area or call 1–800–662-HELP (4357).

^¶^ https://www.cdc.gov/flu/prevent/vaccinations.htm.

** https://www.cdc.gov/mmwr/preview/mmwrhtml/mm6337a4.htm?s_cid.

Outpatient management of suspected EVALI might be considered on a case-by-case basis for patients who are clinically stable, have less severe injury, and for whom follow-up within 24–48 hours of initial evaluation can be assured. Candidates for outpatient management should have normal O_2_ saturation (≥95%), reliable access to care, and strong social support systems. For these patients, empiric use of antimicrobials, including antivirals, if indicated, should be considered. Some patients who initially had mild symptoms experienced a rapid worsening of symptoms within 48 hours. In Illinois and Wisconsin, 72% of patients had either an outpatient or emergency department visit before seeking additional medical care that resulted in hospital admission (*3*). Health care providers should instruct all patients to seek medical care promptly if respiratory symptoms worsen.

**Medical treatment.** Corticosteroids might be helpful in treating this injury. Several case reports describe improvement with corticosteroids, likely because of a blunting of the inflammatory response (*3*–*5*). In a series of patients in Illinois and Wisconsin, 92% of 50 patients received corticosteroids; the medical team documented in 65% of 46 patient notes that “respiratory improvement was due to the use of glucocorticoids” (*3*). Among 140 cases reported nationally to CDC that received corticosteroids, 82% of patients improved (Table). However, the natural progression of this injury is not known, and it is possible that patients might recover without corticosteroids or by avoiding use of e-cigarette, or vaping, products. In some circumstances, it would be advisable to withhold corticosteroids while evaluating patients for infectious etiologies, such as fungal pneumonia, that might worsen with corticosteroid treatment. Nevertheless, because the diagnosis remains one of exclusion, aggressive empiric therapy with corticosteroids, antimicrobial, and antiviral therapy might be warranted for patients with severe illness. A range of corticosteroid doses, durations, and taper plans might be considered on a case-by-case basis. Whenever possible, decisions on the use of corticosteroids and dosing regimen should be made in consultation with a pulmonologist.

Early initiation of antimicrobial treatment for community-acquired pneumonia in accordance with established guidelines** should be strongly considered given the overlapping of signs and symptoms in these conditions. During influenza season, health care providers should consider influenza in all patients with suspected EVALI. Antivirals should be considered in accordance with established guidelines.^††^ Decisions on initiation or discontinuation of treatment should be based on specific clinical features and, when appropriate, in consultation with specialists.

**Follow-up from hospital admission.** Patients discharged from the hospital after inpatient treatment for EVALI should have a follow-up visit no later than 1–2 weeks after discharge that includes pulse-oximetry, and clinicians should consider repeating the CXR. Additional follow-up testing 1–2 months after discharge that might include spirometry, diffusion capacity testing, and CXR should be considered. Long-term effects and the risk of recurrence of EVALI are not known. Whereas many patients’ symptoms resolved, clinicians report that some patients have relapsed during corticosteroid tapers after hospitalization, underscoring the need for close follow-up (personal communication, Lung Injury Response Clinical Working Group, October 2, 2019). Some patients have had persistent hypoxemia (O_2_ saturation <95%), requiring home oxygen at discharge and might need ongoing pulmonary follow-up. Patients treated with high-dose corticosteroids might require care from an endocrinologist to monitor adrenal function.

It is unknown if patients with a history of EVALI are at higher risk for severe complications of influenza or other respiratory viral infections if they are infected simultaneously or after recovering from lung injury. Health care providers should emphasize the importance of annual vaccination against influenza for all persons >6 months of age, including patients with a history of EVALI. In addition, administration of pneumococcal vaccine should be considered according to current guidelines.^§§^

**Addressing exposures.** Advising patients to discontinue use of e-cigarette, or vaping, products should be an integral part of the care approach during an inpatient admission and should be re-emphasized during outpatient follow-up. Cessation of e-cigarette, or vaping, products might speed recovery from this injury; resuming use of e-cigarette, or vaping, products has the potential to cause recurrence of symptoms or lung injury. Evidence-based tobacco product cessation strategies include behavioral counseling and FDA-approved cessation medications.^¶¶^ For patients who have addiction to THC-containing or nicotine-containing products, cognitive-behavioral therapy, contingency management, motivational enhancement therapy, and multidimensional family therapy have been shown to help, and consultation with addiction medicine services should be considered (*8*–*10*).

**Special considerations for groups at high risk.** Patients with certain characteristics or comorbidities, including older age, history of cardiac or lung disease, or pregnancy, might be at higher risk for more severe outcomes. Among reported cases (Table), patients aged >50 years experienced the highest percentage of endotracheal intubation and mechanical ventilation (54%) and the longest mean inpatient stays (15 days). The mean first recorded O_2_ saturations among those who did and did not require intubation were 87% and 92%, respectively (data not shown). Among those with and without past cardiac disease, 31% and 21%, respectively, required intubation (Table). Special consideration might need to be given to patients aged >50 years, because these patients might require longer duration of hospitalization and have a higher risk of intubation (Figure). Rapid identification of exposure, a high index of suspicion of EVALI, initiation of corticosteroids, and specialist consultations might be lifesaving in this patient population.

**FIGURE:**
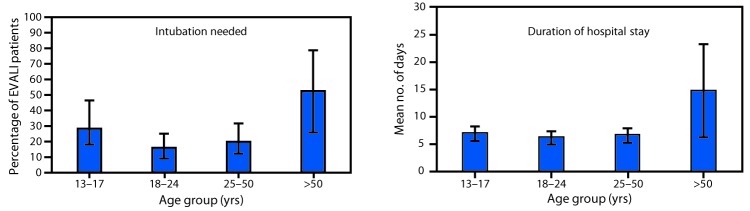
Percentage of persons needing intubation (N = 338) and hospitalization (N = 242) among patients with e-cigarette, or vaping, product use associated lung injury (EVALI), by age of patient — United States, February 1–October 3, 2019*^,†^<Fig_Large></Fig_Large> **Abbreviation:** E-cigarette = electronic cigarette. * Data reported through October 3, 2019, from the following 29 states: Alabama, Delaware, Georgia, Hawaii, Idaho, Illinois, Indiana, Iowa, Kansas, Kentucky, Maine, Maryland, Minnesota, Mississippi, Missouri, Montana, Nevada, New Jersey, New Mexico, Oklahoma, Oregon, Rhode Island, South Carolina, South Dakota, Texas, Vermont, Washington, West Virginia, and Wisconsin. ^†^ 95% confidence intervals indicated by error bars.

Additional data might identify other groups at high risk, provide important information about disparities in outcomes, and help guide clinical care. Certain patients, such as adolescents and young adults, might benefit from specialized services, such as addiction treatment services and providers who have experience with counseling and behavioral health follow-up.

## Clinical Care and Public Health Recommendations

Reporting cases to state, local, territorial, or tribal health departments is critical for accurate surveillance of EVALI. Reporting cases and obtaining and sending products, devices, and clinical and pathologic specimens for testing, can help health departments and CDC determine the cause or causes of these lung injuries.*** CDC is developing *International Classification of Diseases, Tenth Edition, Clinical Modification* coding guidance for health care encounters related to EVALI. Updates, when available, can be found at https://www.cdc.gov/lunginjury (Box 3).

BOX 3 **Clinical Care and Public Health Reporting of e-cigarette, or vaping, product use associated lung injury (EVALI)**

**Considerations at points of care**

- Examples include emergency departments, urgent care, doctors’ offices, etc.
- Consider posting reminders or signage to encourage conversation between patients and providers about use of e-cigarette, or vaping, products.*
- Report cases of lung injury associated with use of e-cigarette, or vaping, products within the past 90 days to state or local health department.
- Determine whether any remaining product, including devices and liquids, is available for testing. Testing can be coordinated with health departments.
- CDC is developing International *Classification of Diseases, Tenth Edition, Clinical Modification* (ICD-10-CM) coding guidance for healthcare encounters related to EVALI. Updates, when available, will be at https://www.cdc.gov/lunginjury.

**Clinical specimen testing by CDC^†^**

- Consider submission of any collected specimens, including bronchoalveolar lavage, blood, or urine, to CDC for evaluation.

**Testing of pathologic specimens by CDC^§^**

- If a lung biopsy or autopsy is performed on a patient suspected of lung injury related to e-cigarette, or vaping, product use, consider submission of fixed lung biopsy tissues or autopsy tissues to CDC for evaluation.
- Testing can include evaluation for lipids on formalin-fixed (wet) lung tissues that have not undergone routine processing.
- Routine microscopic examination will be performed, as well as infectious disease testing, if indicated, on formalin-fixed (wet) tissues, or formalin-fixed, paraffin-embedded tissue specimens.

* https://www.cdc.gov/tobacco/basic_information/e-cigarettes/severe-lung-disease/healthcare-providers/index.html.

^†^ https://www.cdc.gov/tobacco/basic_information/e-cigarettes/severe-lung-disease/health-departments/index.html.

^§^ https://www.cdc.gov/tobacco/basic_information/e-cigarettes/severe-lung-disease/health-departments/index.html.

**Public health recommendations.** At this time, FDA and CDC have not identified the cause or causes of the lung injuries among EVALI cases, and the only commonality among all cases is that patients report the use of e-cigarette, or vaping, products. This outbreak might have more than one cause, and many different substances and product sources are still under investigation. To date, national and state data suggest that products containing THC, particularly those obtained off the street or from other informal sources (e.g., friends, family members, or illicit dealers), are linked to most of the cases and play a major role in the outbreak (*11*,*12*). Therefore, CDC recommends that persons should not use e-cigarette, or vaping, products that contain THC. Persons should not buy any type of e-cigarette, or vaping, products, particularly those containing THC, off the street. Persons should not modify or add any substances to e-cigarette, or vaping, products that are not intended by the manufacturer, including products purchased through retail establishments.

Given that the exclusive use of nicotine-containing products has been reported by a small percentage of persons with EVALI, and that many persons with EVALI report combined use of THC- and nicotine-containing products, the possibility that nicotine-containing products play a role in this outbreak cannot be excluded. Therefore, at present, CDC continues to recommend that persons consider refraining from using e-cigarette, or vaping, products that contain nicotine. If adults are using e-cigarette, or vaping, products to quit cigarette smoking, they should not return to smoking cigarettes; they should use evidence-based treatments, including health care provider counseling and FDA-approved medications.^†††^ If persons continue to use these products, they should carefully monitor themselves for symptoms and see a health care provider immediately if symptoms develop. Irrespective of the ongoing investigation, e-cigarette, or vaping, products should never be used by youths, young adults, or women who are pregnant. There is no safe tobacco product, and the use of any tobacco products, including e-cigarettes, carries a risk. Therefore, persons who do not currently use tobacco products should not start using e-cigarette, or vaping, products.

This investigation is ongoing. CDC will continue to work in collaboration with FDA and state and local partners to investigate cases and to update guidance, as appropriate, as new data emerges from this complex outbreak.

**Summary**

**What is already known about this topic?**

Forty-nine states, the District of Columbia, and one U.S. territory have reported 1,299 cases of lung injury associated with the use of electronic cigarette (e-cigarette), or vaping, products. Twenty-six deaths have been reported from 21 states.

**What is added by this report?**

Based on the most current data, CDC’s updated interim guidance provides a framework for health care providers in their initial assessment, evaluation, management, and follow-up of persons with symptoms of e-cigarette, or vaping, product use associated lung injury (EVALI).

**What are the implications for public health practice?**

Rapid recognition by health care providers of patients with EVALI and an increased understanding of treatment considerations could reduce morbidity and mortality associated with this injury.

## Footnotes

† https://www.cdc.gov/tobacco/basic_information/e-cigarettes/assets/2019-Lung-Injury-Surveillance-Case-Definition-508.pdf.

§ https://www.aafp.org/afp/2017/0101/p29.pdf; https://depts.washington.edu/dbpeds/Screening%20Tools/HEADSS.pdf.

¶ https://www.atsjournals.org/doi/full/10.1164/rccm.201908-1581ST#readcube-epdf; https://academic.oup.com/cid/article/53/7/e25/424286/.

** https://www.atsjournals.org/doi/full/10.1164/rccm.201908-1581ST#readcube-epdf; https://academic.oup.com/cid/article/53/7/e25/424286/.

†† https://www.cdc.gov/flu/professionals/antivirals/summary-clinicians.htm; https://www.idsociety.org/practice-guideline/influenza/.

§§
https://www.cdc.gov/mmwr/preview/mmwrhtml/mm6337a4.htm?s_cid .

¶¶
https://www.cdc.gov/tobacco/campaign/tips/quit-smoking/index.html?s_cid .

*** https://www.cdc.gov/tobacco/basic_information/e-cigarettes/severe-lung-disease/health-departments/index.html.

††† https://www.aafp.org/dam/AAFP/documents/patient_care/tobacco/pharmacologic-guide.pdf.
